# Comparative mitochondrial and chloroplast genomics of a genetically distinct form of *Sargassum* contributing to recent “Golden Tides” in the Western Atlantic

**DOI:** 10.1002/ece3.2630

**Published:** 2016-12-20

**Authors:** Linda A. Amaral‐Zettler, Nicholas B. Dragone, Jeffrey Schell, Beth Slikas, Leslie G. Murphy, Clare E. Morrall, Erik R. Zettler

**Affiliations:** ^1^ Marine Biological LaboratoryJosephine Bay Paul Center for Comparative Molecular Biology and EvolutionWoods HoleMAUSA; ^2^ Department of Earth, Environmental, and Planetary SciencesBrown UniversityProvidenceRIUSA; ^3^ Sea Education AssociationWoods HoleMAUSA; ^4^ St. George's UniversityGrenadaWest Indies; ^5^ Oekologia Environmental Research & EducationFalmouthMAUSA

**Keywords:** accumulations, chloroplast genome, macroalgae, mitogenome, Sargasso Sea, strandings

## Abstract

Over the past 5 years, massive accumulations of holopelagic species of the brown macroalga *Sargassum* in coastal areas of the Caribbean have created “golden tides” that threaten local biodiversity and trigger economic losses associated with beach deterioration and impact on fisheries and tourism. In 2015, the first report identifying the cause of these extreme events implicated a rare form of the holopelagic species *Sargassum natans* (form *VIII*). However, since the first mention of *S. natans VIII* in the 1930s, based solely on morphological characters, no molecular data have confirmed this identification. We generated full‐length mitogenomes and partial chloroplast genomes of all representative holopelagic *Sargassum* species, *S. fluitans III* and *S. natans I* alongside the putatively rare *S. natans VIII*, to demonstrate small but consistent differences between *S. natans I* and *VIII* (7 bp differences out of the 34,727). Our comparative analyses also revealed that both *S. natans I* and *S. natans VIII* share a very close phylogenetic relationship with *S. fluitans III* (94‐ and 96‐bp differences of 34,727). We designed novel primers that amplified regions of the *cox2* and *cox3* marker genes with consistent polymorphic sites that enabled differentiation between the two *S. natans* forms (*I* and *VIII*) from each other and both from *S. fluitans III* in over 150 *Sargassum* samples including those from the 2014 golden tide event. Despite remarkable gene synteny and sequence conservation, the three *Sargassum* forms differ in morphology, ecology, and distribution patterns, warranting more extensive interrogation of holopelagic *Sargassum* genomes as a whole.

## 1 Introduction

**Figure 1:**
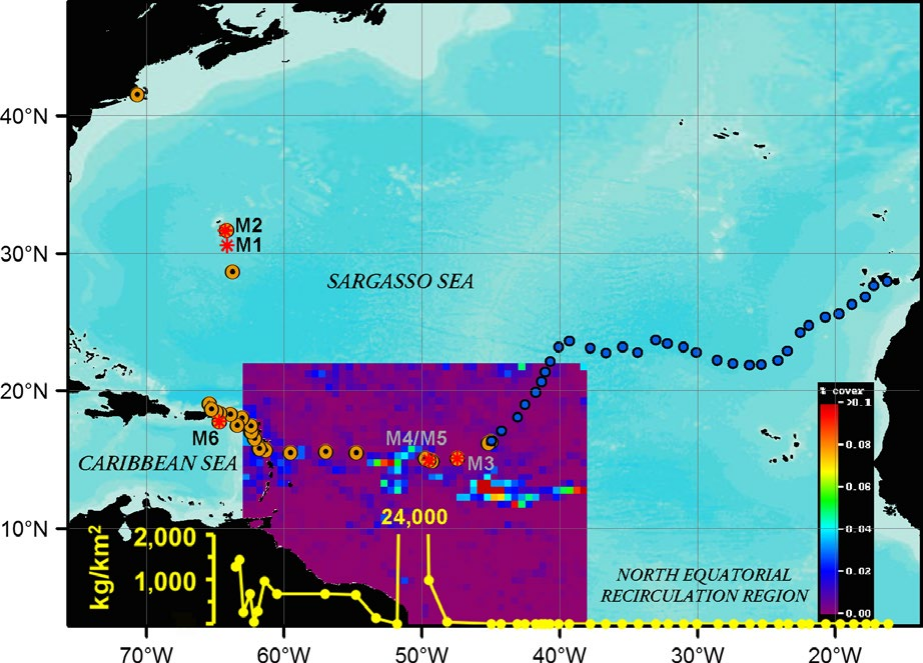
Map of sampling locations where we collected *Sargassum* (yellow dots with black centers), including open‐ocean Atlantic and Caribbean, as well as specimens collected from the shore (Caribbean and Cape Cod, USA). Stations where *Sargassum* was absent from net tows are shown as blue dots, and locations of mitogenome samples as red asterisks. The heatmap that overlies from approximately 38–63°W and 0–22°N depicts estimates of *Sargassum* accumulations from satellite data integrated over the 12‐day period coincident with the timing of the sample collection in that region. The scale of the heatmap at the lower right shows percent of the ocean surface covered by *Sargassum* from 0% to >0.1% (satellite data courtesy of University of South Florida Oceanography Lab, http://optics.marine.usf.edu). The yellow line graph at the bottom of the figure shows *Sargassum* quantity from the cruise net tows, showing the peak around 50°W and good correspondence with estimated densities from the satellite data

The brown alga *Sargassum* is one of the most diverse marine macroalgal genera with 351 recognized species worldwide (Guiry & Guiry, 2016). Members of the genus *Sargassum* are widespread, and most species are benthic with holdfasts, with only two recognized holopelagic species *Sargassum natans* (Linnaeus) Gaillon and *Sargassum fluitans* (Boergesen) Boergesen that do not attach to substrates (*ibid*). These species have gas vesicles and drift and reproduce vegetatively at the surface of the ocean (Dawes & Mathieson, 2008). They are most abundant in the North Atlantic Ocean subtropical gyre, also referred to as the Sargasso Sea (Figure 1), and also occur in the Gulf of Mexico and Caribbean Sea. Holopelagic *Sargassum* has been called the “golden floating rainforest of the Atlantic Ocean” (Laffoley et al., 2011) and functions as an ecosystem engineer that creates a unique floating pelagic biome in substrate‐poor, low‐nutrient open‐ocean waters. The floating *Sargassum* supports over 100 species each of invertebrates and fishes, including ten endemic taxa. *Sargassum* also serves as a nursery habitat for a host of important commercial and threatened species, including large pelagic fish such as tuna and bill fish, and four species of endangered sea turtles (Coston‐Clements, Settle, Hoss, & Cross, 1991).

Although typically found offshore, unusual accumulations of *Sargassum* dubbed “golden tides” (Smetacek & Zingone, 2013), began washing ashore on islands in the Caribbean during 2011 then again in 2014 and 2015, burying beaches, impacting coastal fisheries, restricting harbors, and smothering sea turtle nests (Maurer et al., 2015). Reports of golden tides like those in the Caribbean have also been reported in western Africa and Brazil (De Széchy, Guedes, Baeta‐Neves, & Oliveira, 2012; Smetacek & Zingone, 2013) impacting tourism, food security, and the limited budgets of coastal towns trying to remove the rotting biomass from their beaches. Whether these golden tides represent changes in distribution of existing biomass or result from unusual accumulations due to higher growth rates (“blooms”) has not been established, but the most popular hypothesis is that nutrients supplied by the Amazon and Congo River basins, and also equatorial and coastal upwelling regions along west Africa are allowing fast‐growing *Sargassum* to reach very high concentrations in an area known as the North Equatorial Recirculation Region (NERR, Figure 1), with subsequent flushing toward the Caribbean (Johnson, Ko, Franks, Moreno, & Sanchez‐Rubio, 2013). The impacts of potential “bloom” conditions on the structure of the *Sargassum* populations and the functional diversity of the community of attached and mobile fauna dependent on the *Sargassum* biome are unknown. We also do not know the impacts of the *Sargassum* on coastal ecosystems and the potential for associated fauna to become invasive once the rafts of algae wash ashore.

**Figure 2:**
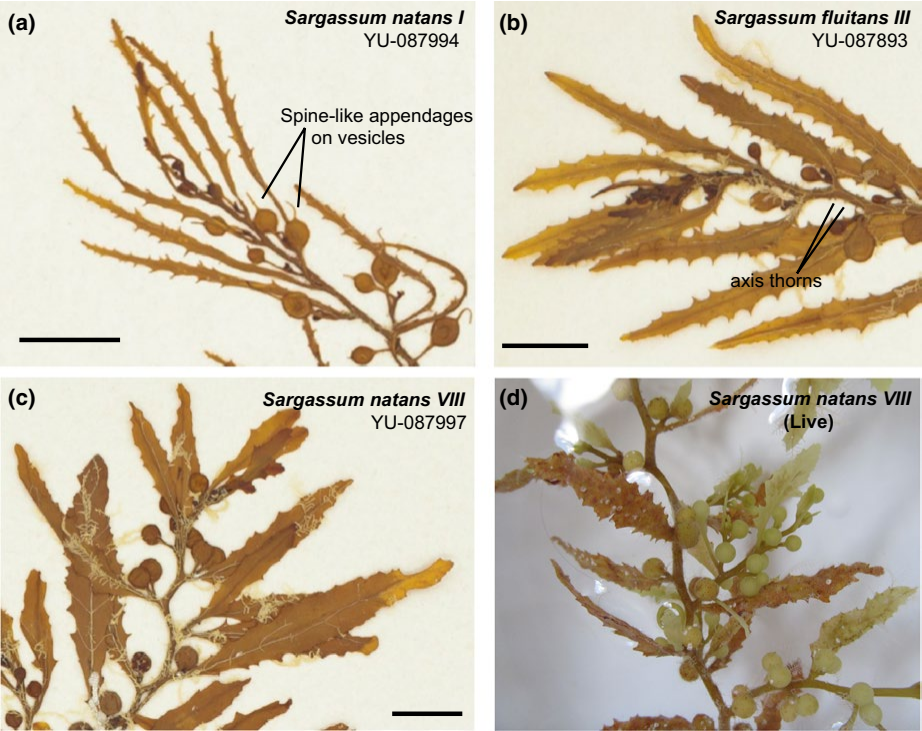
The most common forms of holopelagic *Sargassum* found in Atlantic: (a) herbarium specimen of *Sargassum natans I* from Parr's collection showing characteristic spine‐like appendages on vesicles; (b) herbarium specimen of *Sargassum fluitans III* from Parr's collection showing characteristic axis thorns; (c) herbarium specimen of *S. natans VIII* from Parr's collection showing neither spine‐like appendages nor thorns; (d) live fragment of *S. natans VIII* from sample used for one of the sequenced mitogenomes that matches Parr's morphological description of *S. natans VIII*. Scale bars are 1 cm. Herbarium images a, b, c from the Peabody Museum of Natural History, Yale University (YU); peabody.yale.edu

Only two species of holopelagic *Sargassum* are recognized in contemporary taxonomic guides: *S. natans* and *S. fluitans* with a range historically limited to the Sargasso Sea, Gulf of Mexico, and Caribbean (Figure 2a, b). Extensive collections and early taxonomic work on holopelagic *Sargassum* (Parr, 1939; Winge, 1923) revealed distinct morphological forms of *S. natans* (*I, II, VIII, IX*) and *S. fluitans* (*III, X*). Two forms, *S. natans I* and *S. fluitans III,* proved most abundant in extensive field surveys in the 1930s (Parr, 1939), and despite annual sampling, none of the rarer forms were documented again until the “re‐emergence” of *S. natans VIII* in 2014 (Schell, Goodwin, & Siuda, 2015; Figure 2c, d). These authors noted that during their November 2014 cruise, *Sargassum* concentrations were an order of magnitude higher than previously recorded in their 20‐year data set. Although morphological features identified the accumulating *Sargassum* as the formerly rare form *S. natans VIII*, no genetic analyses have confirmed this observation or Parr's original morphology‐based descriptions.

In order to understand changes in *Sargassum* population structure and potentially mitigate the impact of the *Sargassum* golden tides, marine scientists and managers have to be able to identify the algal species involved and where it is coming from. However, recent efforts to characterize members of the *Sargassum* subgenus *Sargassum* have encountered challenges due to low genetic variability among some clades (Cheang et al., 2010; Cho, Lee, Ko, Mattio, & Boo, 2012; Dixon et al., 2014; Mattio, Bolton, & Anderson, 2015; Mattio et al., 2013) and a lack of marker genes capable of distinguishing between closely related taxa in general (Camacho, Mattio, Draisma, Fredericq, & Diaz‐Pulido, 2014; Mattio & Payri, 2010). Thus, new molecular ecology resources are urgently needed to aid this endeavor. The use of mitogenomes to differentiate between different species of *Sargassum* has recently provided a bit of traction in terms of offering new sets of genetic markers for population studies of *Sargassum* species from the Pacific (Bi & Zhou, 2016; Liu & Pang, 2014; Liu, Pang, & Chen, 2016; Liu, Pang, Li, & Li, 2015; Liu, Pang, & Luo, 2014). In addition to mitochondrial genomes, the first *Sargassum* chloroplast genome from the benthic species *S. horneri* recently became available providing another source of potential marker genes (Liu & Pang, 2015).

Here, we explored the potential for mitogenomes and chloroplast coding regions to distinguish between Atlantic *Sargassum* holopelagic species by generating and comparing complete mitochondrial genomes and partial chloroplast genomes from all of the known holopelagic *Sargassum* species using next‐generation sequencing approaches. From our reference mitogenomes, we developed two marker gene primer sets that are capable of differentiating between all three holopelagic *Sargassum* forms encountered in the Atlantic: *S. natans I, S. natans VIII,* and *S. fluitans III*. Using all *Sargassum* mitogenomes available to date including the six new ones we generated, we also provide a phylogenetic placement for the three forms of *Sargassum* most commonly encountered in the tropical and subtropical seas of the western Atlantic.

## 2 Materials and Methods

### 2.1 Sample collection and DNA extraction

We collected samples for our six reference mitochondrial and partial chloroplast genomes and 155 marker gene validations from 71 independent clumps of *Sargassum* from across the NW Atlantic Ocean and in the Caribbean Sea (see Figure 1 and Table S1 for details). Samples of holopelagic *Sargassum* were collected in towed neuston nets, as well as in dip nets during four separate cruises in 2012, 2014, 2015, and 2016 in the North Atlantic Ocean. Additional samples of clumps that had stranded on shore were collected by hand in 2014 and 2016 (see Table S1).

Clumps of macroalgae were assigned species identifications using morphological characters defined by Parr (1939) including frond characteristics, the presence or absence of thorns on the axes, and spine‐like appendages on the air bladders (vesicles), as well as by blade (leaf) size. According to Parr's definitions, *S. fluitans III* can be distinguished from both *S. natans I* and *S. natans VIII* based on the presence of thorns on its axes. *S*. *fluitans* also does not have spine‐like appendages on its vesicles that are usually present on vesicles of *S. natans I* and occasionally found on vesicles of *S. natans VIII*. The two species of *S. natans* can also be distinguished based on their blade width. *S. natans I* has blades that are much narrower than *S. natans VIII*. Figure 2 depicts museum vouchers for Parr's original species morphological descriptions of the different forms sequenced in this study.

After identifying a particular sample, we removed 5 cm of the axis from each individual clump, with all attached blades and vesicles, for processing and preservation. In the laboratory, samples were rinsed in 0.22‐μm‐filtered seawater, shaken, blotted with a paper towel to remove the excess water, and then placed in individual plastic bags containing 15 g of silica gel (Activa Products, Marshall, TX, USA). The fronds became fully desiccated after 24 hr in the silica gel.

Samples for molecular characterizations included those preserved in silica gel and others in RNAlater^**®**^ (ThermoFisher, Waltham, MA, USA). Voucher material has been archived for all the samples in the Amaral‐Zettler laboratory and is available for viewing upon request. RNAlater^**®**^ sample extraction followed protocols of Zettler, Mincer, and Amaral‐Zettler (2013). For *Sargassum* preserved in silica gel, we used a modified Qiagen DNeasy Plant Mini‐kit protocol (Qiagen, Valencia, CA, USA) to extract genomic DNA. The modified protocol followed the manufacturer's instructions with a few changes. In place of step one of the Qiagen protocol, we crushed approximately 0.05 g of preserved *Sargassum* tissue in an individual 1.5‐ml microfuge tube with a mini‐pestle (USA Scientific, Ocala, FL) to prepare the sample for extraction. In step two, after reagents were added, the samples were vortexed on high speed for 30 s and then shaken in a vortex mixer with a 24‐tube holder for 3 min. In step nine, the columns were spun at 14,000 rpm for 3 min to remove any residual buffer. Finally, the elutions in steps 11 and 12 occurred with 50 μl of buffer AE, for a total elution volume of 100 μl. All extracted genomic DNA was cleaned using the MoBio PowerClean Pro DNA Clean‐Up Kit (MoBio, Carlsbad, CA, USA), following manufacturer's instructions.

### 2.2 Library construction and sequencing

We constructed genomic libraries for representative samples of each *Sargassum* form (number of individuals sequenced in parentheses): *S. natans VIII* (three specimens); *S. natans I* (one specimen); and *S. fluitans III* (two specimens). Table S1 and Figure 1 (M1–M6) provide the GPS coordinates and locations where the samples used in metagenomic library constructions originated. We prepared short (175 bp) and/or long (~250–450 bp) insert libraries by shearing the genomic DNAs to the corresponding lengths on the Covaris S220 (Covaris, Inc., Woburn, MA, USA) and constructed genomic libraries using Ovation Ultralow DR Multiplex Systems 9–16 kits (NuGEN Technologies Inc., San Carlos, CA, USA). We sequenced samples on either a HiSeq 1000 or NextSeq 500 Illumina platform according to manufacturer protocols. Table S1 contains specific details on library preparation and sequencing performed for each sample.

### 2.3 Bioinformatics and phylogenetics

Resulting Illumina raw reads were merged and quality‐checked using a series of Python scripts: “iu‐merge‐pairs” with the “enforce‐Q30‐check” option, to remove low‐quality reads and merge pairs, followed by “iu‐filter‐merged‐reads” to retain only merged pairs with no mismatches in the overlapping region (Minoche, Dohm, & Himmelbauer, 2011). We used CLC genomic workbench to assemble and map reads back to single mitochondrial genomic contigs (http://www.clcbio.com) and Geneious v. 8.0.5 (Biomatters, Ltd, Aukland, New Zealand) for gene annotation and visualization of the mitogenomes by importing annotation tracks from existing sequenced mitogenomes of other *Sargassum* species in GenBank. We used a similar strategy for mapping chloroplast gene reads back to the *S. horneri* chloroplast genome in order to access variation of polymorphic sites in the chloroplast coding regions of our representative *Sargassum* species.

For our phylogenetic analyses of mitogenomes, we removed any positions of uncertain homology in the mitochondrial alignments and inferred phylogenetic trees using 34,290 homologous nucleotide positions in RAxML version 7.2.8 (Stamatakis, 2006) and a general time reversible substitution model with gamma and invariant sites implemented through Geneious. One thousand bootstrap replicates determined the confidence of the branch support in the resulting phylogeny.

### 2.4 Confirmation of polymorphic sites via PCR

To assess whether the variant sites within mitochondrial genomes that we discovered were fixed, we designed primers that targeted the relevant cytochrome oxidase subunit 2 (*cox2*) and cytochrome oxidase subunit 3 (*cox3*) gene regions and obtained sequences for 71 geographically diverse individuals, including representatives of each holopelagic *Sargassum* form (number of individuals sequenced in parentheses): (*S. natans VIII* (*n* = 53); *S. natans I* (*n* = 13); and *S. fluitans III* (*n* = 5): See Supplementary Online Material for details, different parts of some individuals were extracted and sequenced more than once). Confirmation of polymorphic sites differentiating our three *Sargassum* mitogenomes was accomplished using primers targeting *cox2* (*cox2*‐370F: 5′‐CAAAGATGGATTCGACGGTTGG‐3′, *cox2*‐776R: 5′‐CCGGTATCAAACTCGCCCTT‐3′) and *cox3* (*cox3*‐467F: 5′‐GGTTCAACGACACCCATTT‐3′, *cox3*‐901R: 5′‐TAGCGTGATGAGCCCATG‐3′) gene regions. PCR was carried out in triplicate 25 μl reactions with 1× NEB OneTaq (New England BioLabs, Ipswich, MA, USA), 1 μl of 10 mmol/L forward primer, and 1 μl of 10 mmol/L reverse primer. Cycling conditions for both primer sets were as follows: an initial 94°C denaturation step for 4 min; 30 cycles of 94°C for 1 min, 50°C for 30 s, 72°C for 1 min; and a final 7‐minute extension at 72°C. The triplicate PCRs were pooled after amplification and purified using MinElute PCR Purification spin columns (Qiagen). Purified DNA was eluted in 10 μl of MinElute buffer EB. Cleaned PCR products were amplified for 60 cycles (96°C for 10 s, 50°C for 5 s, 60°C for 4 min) with BigDye at 1/16th concentration and sequenced on an ABI 3730XL (Applied Biosystems, Foster City, CA, USA) capillary sequencer at the W. M. Keck Ecological and Evolutionary Genetics Facility at the Marine Biological Laboratory.

## 3 Results

**Figure 3:**
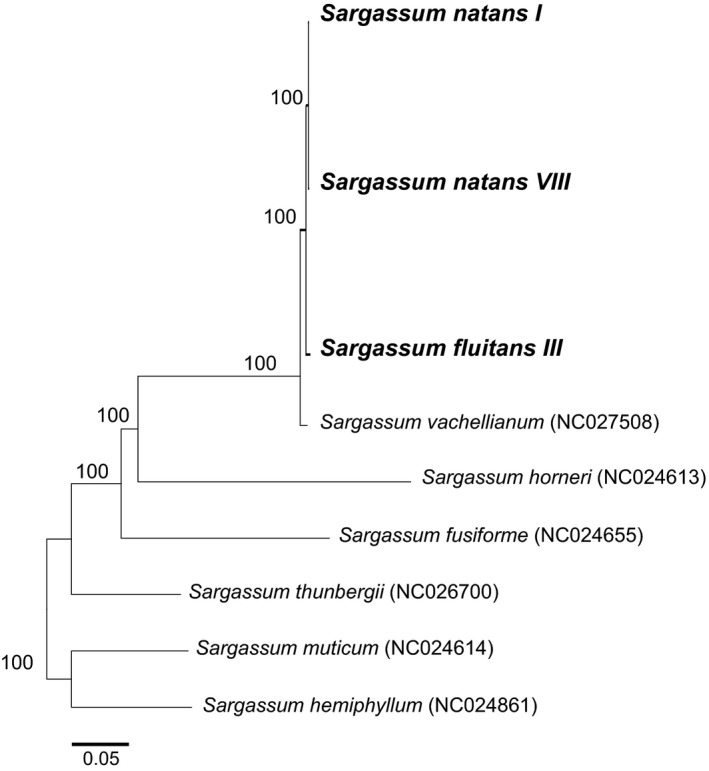
A maximum‐likelihood‐inferred phylogeny of mitogenomes from all available *Sargassum* species alongside the holopelagic species sequenced in this study (in bold). Scale bar represents evolutionary distance, and numbers at the nodes represent bootstrap confidence values. GenBank numbers follow the names of published mitogenomes

Our phylogenetic analyses based on mitochondrial sequence data revealed three genetically distinct forms of holopelagic *Sargassum*. The holopelagic *Sargassum* responsible for the golden tides in the Caribbean fell into the *S. natans* species complex with high bootstrap support (100%) and was not *S. fluitans* as often reported. Figure 3 depicts a maximum‐likelihood phylogenetic tree based on complete mitochondrial genomes of the three holopelagic *Sargassum* forms and six additional *Sargassum* species (representing all the available *Sargassum* mitogenomes in GenBank). All three holopelagic *Sargassum* mitogenomes are 34,727 bp in length and share identical gene synteny and content over the entire mitogenome (65 genes), as well as a similar AT content (63.8%). At the nucleotide level, the two *S. natans* forms differed at only seven sites of 34,727 bp (Table 1; differences are highlighted between the two *S. natans* forms), and *S. natans I* and *S. natans VIII* differed from *S. fluitans III* at 93 and 96 sites, respectively (Table 1). For the two *S. natans* mitogenomes, differences occurring in coding regions resulted in amino acid differences in five protein‐coding genes (ribosomal protein L5 (*rpl5*)*,* ribosomal protein S19 (*rps19*)*,* ribosomal protein S13 (*rps13*)*,* cytochrome oxidase 3 (*cox3*)*,* and NADH dehydrogenase subunit 6 (*nad6*)). We also found a single polymorphism across the two *S. natans* forms in both the 23S ribosomal RNA (*23S rRNA*) and *16S rRNA* genes. The three holopelagic *Sargassum* differed at 370, 372, and 383 sites from the closest species with a sequenced mitogenome—*S. vachellianum*, a benthic *Sargassum* species endemic to China.

**Table 1:** Nucleotide differences and corresponding amino acid changes across the complete mitogenomes of the three holopelagic *Sargassum* forms

Genomic position	Locus	Nucleotide			Amino acid
		*Sargassum fluitans III*	*Sargassum natans I*	*S. natans VIII*	
1279	*23S rRNA*	C	T	T	–
1883	*23S rRNA*	C	T	C	–
2658	*23S rRNA*	A	G	G	–
2662	Intergenic Region	C	T	T	–
2688	Intergenic Region	C	T	T	–
2691	Intergenic Region	G	T	T	–
2714	Intergenic Region	G	T	T	–
2715	Intergenic Region	A	T	T	–
2716	Intergenic Region	T	A	A	–
2717	Intergenic Region	T	A	A	–
2718	Intergenic Region	A	T	T	–
2719	Intergenic Region	A	C	C	–
4629	*rpl6*	C	T	T	Ala > Ala
4928	*rps2*	C	T	T	Pro > Pro
5147	*rps2*	C	T	T	Thr > Thr
5424	*rps4*	G	A	A	Ala > Ala
5470	*rps4*	T	C	C	Leu > Leu
5491	*rps4*	A	G	G	Ile > Val
5832	*rps4*	C	T	T	Pro > Pro
6097	Intergenic Region	T	C	C	–
6805	*nad1*	T	C	C	Val > Val
7033	*nad1*	C	T	T	Arg > Arg
7237	*tatC*	T	A	A	Ile > Phe
7720	*tatC*	G	A	A	Leu > Leu
7837	Intergenic Region	A	T	T	–
7903	*trnW(cca)*	A	G	G	–
8090	*orf39*	T	C	C	Phe > Ser
8250	*trnQ(uug)*	G	A	A	–
8438	*rps12*	G	A	A	Ala > Thr
8857	*rps7*	A	G	G	Ser > Gly
9030	*rps7*	A	T	T	Ile > Ile
9153	*rps7*	A	G	G	Ala > Ala
9863	*rpl14*	C	A	A	Val > Val
10018	*rpl14*	C	T	T	Ser > Leu
10313	*rpl5*	C	C	T	Pro > Ser
10783	*orf129*	C	A	A	Glu > Stop
12021	*rps3*	A	T	T	Ser > Ser
12429	*rps19*	G	G	T	Gln > Lys
12821	*rpl2*	T	C	C	Gly > Gly
12878	*rpl2*	G	T	T	Ala > Ala
13502	*rps13*	G	G	T	Ala > Ser
13537	*rps13*	T	C	C	Phe > Phe
13583	*rps13*	G	C	C	Val > Leu
14111	*rps11*	T	C	C	Val > Val
14466	*cox3*	A	G	G	Leu > Leu
14516	*cox3*	G	A	G	Gly > Asp > Gly
15108	*cox3*	G	A	A	Gly > Gly
15249	Intergenic Spacer	G	C	C	–
17135	*nad2*	C	T	T	Val > Val
18237	*cox1*	T	C	C	Phe > Phe
18645	*cox1*	G	T	T	Thr > Thr
19527	Intergenic Spacer	T	C	C	–
19698	*nad9*	T	C	C	Tyr > His
20527	*cob*	T	C	C	Gly > Gly
21649	*cox2*	A	G	G	Leu > Leu
21693	*cox2*	C	G	G	Thr > Ser
21780	*cox2*	G	C	C	Gly > Ala
21940	*cox2*	A	G	G	Leu > Leu
22037	*cox2*	C	T	T	Leu > Leu
22216	*cox2*	A	T	T	Ser > Ser
22453	*cox2*	C	T	T	Thr > Thr
22469	*cox2*	G	A	A	Glu > Lys
22474	*cox2*	T	C	C	Ser > Ser
23156	*cox2*	C	T	T	Pro > Ser
23290	*cox2*	G	A	A	Val > Val
23294	*cox2*	T	G	G	Tyr > Asp
23577	*cox2*	G	A	A	Arg > Lys
23856	*cox2*	T	C	C	Leu > Pro
24568	*cox2*	T	A	A	Asp > Glu
24665	*nad4*	G	A	A	Gly > Glu
24706	*nad4*	T	G	G	Ser > Ala
24861	*nad4*	C	T	T	Thr > Thr
26136	*trnl(uau)*	T	A	A	–
26272	*nad5*	T	C	C	Cys > Cys
26536	*nad5*	C	T	T	Phe > Phe
26926	*nad5*	A	T	T	Val > Val
26962	*nad5*	T	A	A	Ala > Ala
27508	*nad5*	C	T	T	Pro > Pro
28323	*nad6*	G	A	A	Ala > Ala
28444	*nad6*	T	T	A	Phe > Ile
28463	*nad6*	C	G	G	Thr > Ser
28656	*nad6*	C	T	T	Thr > Thr
28890	*nad6*	A	G	G	Leu > Leu
28895	*nad6*	T	G	G	Leu > Trp
29053	*nad11*	T	C	C	Asn > Asn
29299	*nad11*	A	T	T	Leu > Phe
29518	*nad11*	C	T	T	Cys > Cys
29976	*rps14*	G	A	A	Ser > Asn
30082	*rps14*	G	A	A	Arg > Arg
30958	*rps10*	T	A	A	Leu > Leu
31048	*rps10*	C	T	T	Ser > Ser
31120	*rps10*	G	C	C	Lys > Asn
31578	Intergenic Spacer	T	A	A	–
31920	*16S rRNA*	A	A	C	–
32415	*16S rRNA*	C	T	T	–
33027	*16S rRNA*	G	A	A	–
33828	*nad7*	C	A	A	Leu > Leu
34589	*nad7*	G	A	A	Stop > Stop

Shaded areas highlight differences between *S. natans I* and *VIII*.

Sequencing partial *cox2* and *cox3* genes for 71 different holopelagic *Sargassum* individuals revealed no polymorphisms within forms at the nucleotide sites that differentiated the two *S. natans* from each other or at sites that distinguished both *S. natans* from *S. fluitans III*; that is, the differences between forms and species appear to be fixed. Further interrogating our genomic datasets, we also could not detect differences between the three *Sargassum* forms using barcoding genes often used in phylogenetic or population genetic studies: nuclear genes *18S rRNA*, Internal Transcribed Spacer 2 (*ITS‐2)* and *5.8S rRNA,* or chloroplast genes ribulose‐1,5‐bisphosphate carboxylase/oxygenase large subunit—intergenic spacer—ribulose‐1,5‐bisphosphate carboxylase/oxygenase small subunit (*rbcL*—intergenic spacer—*rbcS*). Both nuclear *18S* and *5.8S rRNA* genes were identical across the forms, while the intergenic spacer between *rbcL* and *rbcS* and three sites in the *rbcL* gene distinguished *S. fluitans* from *S. natans*, but could not differentiate between the two *S. natans* forms. *ITS‐2* genes for *S. natans I* and *S. fluitans III* were identical, while *S. natans VIII* showed a single bp difference. Across the gold‐standard barcoding gene, *cox1*,* S. fluitans* differed from the *S. natans* forms at only two sites, and the two *S. natans* forms were identical.

In addition to comparisons between mitogenomes and nuclear marker genes used in other studies targeting *Sargassum*, we also compared the chloroplast coding regions that we were able to recover from mapping reads from our six representative samples against the full‐length chloroplast genome of *S. horneri* as a reference (Liu & Pang, 2015). Near‐complete chloroplast genomes were recovered from one of the *S. natans VIII* samples (C6) and the *S. natans I* sample (C2) and included all of the coding regions reported in *S. horneri*. Table S2 lists all the coding regions found in *S. horneri* and their status (i.e., level of completeness) in our reference *Sargassum* samples. Polymorphic sites occurred in the following *S. natans I* and *S. natans VIII* genes (indicated with a number in Table S2 corresponding to a polymorphic location in a gene): light‐independent protochlorophyllide reductase subunit B gene (*chlB)*,* clpC*, conserved hypothetical gene (*orf 219)*, ribosomal protein L2 (*rpl2)*, DNA‐directed RNA polymerase subunit beta (*rpoB)*, DNA‐directed RNA polymerase subunit C1 (*rpoC1)*, DNA‐directed RNA polymerase subunit C2 (*rpoC2)*, thiazole synthase (*thiG)*, and conserved hypothetical gene (*ycf19)* coding regions. Additional polymorphic sites were detected in the *23S rRNA* gene, as well as the tRNA coding region for *tRNA‐Threonine* and one copy of the *tRNA‐Methionine*. In addition to these variable sites—we discovered candidate polymorphisms that may reveal population‐level differences in the Photosystem I iron–sulfur center (*psaC*:* S. fluitans III*), *23S rRNA* (*S. natans VIII*), and *tRNA‐Isoleucine* (*S. fluitans III*) genes, but these particular variations occurred in regions of low coverage and thus need to be confirmed with additional sequencing. Overall, we detected 12 genes that contained variable regions between *S. natans VIII* and *S. natans I* samples, 17 genes between *S. natans VIII* and *S. fluitans III*, and 19 genes between *S. natans I* and *S. fluitans III* (Table S2).

For chloroplast genomes that were most complete *S. natans VIII* (C6) and the *S. natans I* (C2), gene synteny and content were identical to *S. horneri* with 173 genes (Table S2), including six coding for rRNA genes (two copies each) and 28 coding for tRNA genes (with multiple copies of some, designated with a “1” or a “2” in Table S2). As described extensively in Liu and Pang (2015), there is a high degree of conservation in chloroplast DNA among the brown algae and our results were consistent with this finding.

## 4 Discussion

Despite massive strandings of *Sargassum* on beaches in the Caribbean, Brazil, and Africa during the last several years, it has not been clear what species are involved in these events or where the accumulations originated. We sampled from beaches and waters of the Caribbean Sea, as well as from large accumulations in the open Atlantic, where our net tows revealed unusually high concentrations of over 24,000 kg/km^2^; satellite data supported the idea that concentrations we sampled were part of a larger accumulation that extended from the Caribbean toward the NERR (Figure 1). Our molecular results confirmed that *S. natans VIII* is causing the large accumulations and associated strandings (a.k.a. golden tides) and that it is genetically distinct from the *S. natans I* and *S. fluitans III* reported from the Atlantic in the past (Schell et al., 2015). Based on morphological features of archived specimens of Parr's original type material, we confirmed that the accumulating form encountered in 2014 and 2015 is the formerly rare *S. natans VIII*, not the better‐known *S. fluitans III* that is morphologically similar. *S. natans VIII* was the dominant form of *Sargassum* collected in 2014 and 2015 from cruises and beach collections used in our molecular analyses (Figure 1).

Taxonomic problems within the genus *Sargassum* C. Agardh are broadly reported in the literature due to the morphological variability of the genus and the fact that standard marker genes (*ITS2*,* rbcLS*,* cox3, mtsp,* etc.) fail to resolve relationships between the closely related members of the holopelagic species. While *cox3* was previously suggested as a possible marker to delineate *Sargassum* species (Camacho et al., 2014), existing published primers target a portion of this gene that fails to differentiate between the holopelagic members of the subgenus *Sargassum* including *S. natans VIII* responsible for the recent strandings. As limited information is available from public databases for the regions of the *cox2* and *cox3* genes covered by our new primers, it is difficult to predict their utility to differentiate between other *Sargassum* subgenera. However, our *cox3* primer set has an identical match to *S. vachellianum,* for example, so may be able to differentiate between other related benthic species. Limited application of our *cox3* primers to benthic *Sargassum* spp. revealed that the primers may also differentiate between benthic *Sargassum* spp. from the Caribbean (Amaral‐Zettler, unpublished observation). Our *cox2* primers, on the other hand, have at least three mismatches in either or both the forward or reverse priming region in benthic *Sargassum* spp. with available *cox2* genes for comparison, so are unlikely to be useful for population studies beyond the holopelagic forms.

Due to limitations in using single marker genes for comparative brown algal phylogenetic studies, investigators are increasingly turning to comparative genomics of organelles as tools to differentiate between closely related taxa. Our study is the first to demonstrate genetic variability between the different morphotypes of *Sargassum* originally described in the first part of the 20th century. The fact that we were able to confirm that the species involved in the “golden tides” are a genetically distinct and formerly rare form of holopelagic *Sargassum* is important because it suggests that the absolute and relative abundance of the different forms of *Sargassum* is shifting in the NW Atlantic and Caribbean Sea. While the reasons for this shift are unknown, our findings support the existence of a formerly overlooked, genetically distinct population from the NERR that is increasing in abundance and expanding into other areas of the tropical Atlantic.

Since 2011, several groups have tried to model potential sources of the emerging *Sargassum* accumulations based on known distributions of *S. natans* and *S. fluitans* in the Sargasso and Caribbean Seas. Johnson et al. (2013) could not trace a Sargasso Sea source for the Caribbean strandings using satellite‐tracked drifters, but using back‐tracking models argued for a source in the NERR. Likewise, modeling work of Gower, Young, and King (2013) suggested a separate origin to the 2011 *Sargassum* strandings distinct from earlier satellite detections in 2005 and 2003‐2005 where accumulations were localized to the Gulf of Mexico. As satellite observations cannot differentiate between the distinct species of *Sargassum*, much confusion originated from these original publications regarding the probable source populations responsible for the accumulations. Our results, coupled with the modeling results, as well as visual sightings of large mats of *Sargassum* in the South Atlantic off of Africa and Brazil (De Széchy et al., 2012) support the hypothesis that a genetically distinct population of *S. natans VIII* always occurred in the NERR but never experienced the proper set of conditions to form large accumulations like the ones that are causing the recent strandings. Parr (1939) consistently reported *S. natans VIII* from the Caribbean, but always in small amounts. Our confirmation of a distinct population and the scale of recent strandings pose the questions: *What is causing this previously rare taxon to expand its range and dominate areas where it was formerly rare? Are we witnessing a regime shift in the NERR and North Atlantic ecosystems?*

The ability to accurately identify the type of *Sargassum* is important as we start to address these questions, but recent publications disagree on morphological characters that differentiate between *Sargassum* forms (De Széchy et al., 2012), such as the presence or absence of thorns on the axes as a character differentiating *S. fluitans* from *S. natans* (see Schell et al., 2015: Figure 1b vs. De Széchy et al., 2012: Figure 4). *Sargassum fluitans III* has a range that overlaps *S. natans VIII* and the two can be readily confused; even with considerable expertise of the scientists identifying our samples using morphological characters alone, our molecular results showed that some specimens had been misclassified, highlighting the importance of genetic analyses to confirm the identity of these closely related species. The primers and methods described in this study address this need by providing accurate and nonsubjective identification of the holopelagic species.

Despite consistent differences at the mitogenome and marker gene levels, our molecular results demonstrate that overall, the holopelagic *Sargassum* species exhibit remarkable genetic similarity. Likewise, differences in chloroplast coding regions are modest at best. In their comparative chloroplast genomics of four brown algae including *S. horneri*,* Fucus vesiculosus*,* Saccharina japonica*, and *Ectocarpus siliculosus*, Liu and Pang (2015) found that despite a smaller chloroplast genome size, *S. horneri* shared all the same genes and gene order (synteny) as *F*. *vesiculosus*. A list of all the coding regions in the chloroplast genome for *S. natans I* and *VIII* can be found in Table S2. Deeper sequencing is required to interrogate the variation in intergenic spacer regions of holopelagic *Sargassum* chloroplast genomes, as well as nuclear genomes overall and should be the focus of future efforts.

Recent studies report temporal changes in the richness of *Sargassum*'s attached and mobile faunal communities (Huffard, Von Thun, Sherman, Sealey, & Smith, 2014) and in the relative proportions of the different forms of the two dominant *Sargassum* species *S. fluitans* and *S. natans* (Schell et al., 2015). We do not know what is causing these changes, whether it is part of a natural cycle or represents a shift in open‐ocean ecosystems due to anthropogenic impacts such as nutrient input and climate change. Something is leading to increased abundance and dominance of *S. natans VIII*, a form that was previously rare, and there is evidence that the different forms have different ecologies and associated fauna (Schell et al., 2015). This suggests that the changes in *Sargassum* populations and distribution will have corresponding impacts on both open‐ocean communities and the coastal regions where golden tides occur. Understanding of the holopelagic *Sargassum* ecosystem and environmental costs of recent and future golden tide events cannot proceed without proper species identification of the *Sargassum* involved. Our PCR‐screening approach of *cox2* and *cox3* genes offers a straight‐forward means of confirming morphological identifications of the holopelagic *Sargassum* species.

This study confirms that not only the amount, but also the population structure of *Sargassum* is changing and that recent *Sargassum* strandings represent a major biological shift in open‐ocean and coastal ecosystems around the Atlantic. Our study identifies the *Sargassum* responsible as a formerly rare form and generates important questions about the diversification of holopelagic species possessing such limited variability at the genomic scales documented in our study: *Do they represent recently diverged lineages?* The case of *Sargassum* is interesting in that, despite clear morphological, ecological, and distribution differences, the evolutionary distances between *S. natans* and *S. fluitans* observed in our phylogenetic analysis are very small compared to their benthic counterparts and suggest that they are relatively new species. Current theories argue that human‐mediated environmental changes such as increases in temperature and nutrient loads are leading to blooms of previously “rare” genotypes already existing in a population. The increase in abundance along with asexual reproduction and gas vesicles in the holopelagic forms enhance their ability to be transported long distances and may increase their invasion potential (Paula & Eston, 1987). With the new repertoire of genetic information now available through our efforts, we are poised to expand our understanding of population‐level variation in holopelagic *Sargassum* and its associated taxa.

## Conflict of Interests

We have no competing interests.

## Data Accessibility

GenBank accessions are KY084907–KY084912 for *Sargassum* mitochondrial genomes. KY126998–KY127307 for *cox2* and *cox3* population sets and KY206015–KY206739 for chloroplast genes from *Sargassum* references.
