# Supplementary material for: Comparative mitochondrial and chloroplast genomics of a genetically distinct form of Sargassum contributing to recent “Golden Tides” in the Western Atlantic
Source: Ecol Evol. 2016 Dec 20;7(2):516–25. doi: 10.1002/ece3.2630 (PMC5243773; doi:10.1002/ece3.2630)
Supplement: Supplementary file 2 [file ECE3-7-516-s002.docx]

Table S2. Nucleotide differences across chloroplast genes of the three holopelagic *Sargassum* forms. (Multiple numbers in a given cell indicate multiple polymorphic sites; * = the nucleotide at that site is identical to those in the other libraries; X = incomplete gene content, not considered in comparison). Chloroplast genomes (C1-C6) match mitogenome designations (see Table S1).

|  |  | | *S. natans VIII*  (SN8) | | | *S. natans I*  (SN1) | *S. fluitans III*  (SF3) | |  | Differentiating Polymorphic Sites | | | | |
| --- | --- | --- | --- | --- | --- | --- | --- | --- | --- | --- | --- | --- | --- | --- |
| Gene | Function | | C3 | C4 | C6 | C2 | C5 | C1 |  | SN8 | SF3 | SN8 vs.  SN1 | SN8 vs.  SF3 | SN1 vs. SF3 |
| *acsF* | Light harvesting and chl biosynthesis | | X | * | * | * | X | X |  |  |  |  |  |  |
| *atpA* | Electron transport and ATP synthesis | | X | * | * | * | X | X |  |  |  |  |  |  |
| *atpB* | Electron transport and ATP synthesis | | */*/* | */*/* | */*/* | */*/* | X | 1/2/3 |  |  |  |  | √ | √ |
| *atpD* | Electron transport and ATP synthesis | | X | * | * | * | X | X |  |  |  |  |  |  |
| *atpE* | Electron transport and ATP synthesis | | * | * | * | * | X | * |  |  |  |  |  |  |
| *atpF* | Electron transport and ATP synthesis | | X | * | * | * | 1 | X |  |  |  |  | √ | √ |
| *atpG* | Electron transport and ATP synthesis | | X | * | * | * | X | X |  |  |  |  |  |  |
| *atpH* | Electron transport and ATP synthesis | | * | * | * | * | X | * |  |  |  |  |  |  |
| *atpI* | Electron transport and ATP synthesis | | X | * | * | * | X | X |  |  |  |  |  |  |
| *cbbx* | Signal transduction | | X | * | * | * | X | X |  |  |  |  |  |  |
| *ccs1* | Electron transport and ATP synthesis | | X | * | * | * | X | X |  |  |  |  |  |  |
| *ccsA* | Electron transport and ATP synthesis | | X | * | * | * | X | X |  |  |  |  |  |  |
| *chlB* | Light harvesting and chl biosynthesis | | * | * | * | 1 | X | X |  |  |  | √ |  |  |
| *chlI* | Light harvesting and chl biosynthesis | | * | * | * | * | X | * |  |  |  |  |  |  |
| *chlL* | Light harvesting and chl biosynthesis | | X | * | * | * | X | X |  |  |  |  |  |  |
| *chlN* | Light harvesting and chl biosynthesis | | * | * | * | * | X | * |  |  |  |  |  |  |
| *clpC* | Proteolysis | | */*/*/*/* | */*/*/*/* | */*/*/*/* | 1/*/*/4/* | X | 1/2/3/*/5 |  |  |  | √ | √ | √ |
| *dnaB* | Transcription and translation | | X | * | * | * | X | X |  |  |  |  |  |  |
| *dnaK* | Chaperones | | X | * | * | * | X | X |  |  |  |  |  |  |
| *ftrB* | Signal transduction | | X | * | * | * | X | X |  |  |  |  |  |  |
| *ftsH* | Proteolysis | | X | * | * | * | X | X |  |  |  |  |  |  |
| *groEL* | Chaperones | | X | * | * | * | X | X |  |  |  |  |  |  |
| *ilvB* | Carbon assimilation | | X | X | X | * | X | X |  |  |  |  |  |  |
| *ilvH* | Carbon assimilation | | X | * | * | * | X | X |  |  |  |  |  |  |
| *orf219* | Conserved hypothetical genes | | X | * | * | 1 | X | X |  |  |  | √ |  |  |
| *orf467* | Conserved hypothetical genes | | X | X | X | X | X | X |  |  |  |  |  |  |
| *petA* | Electron transport and ATP synthesis | | * | * | * | * | X | 1 |  |  |  |  | √ | √ |
| *petB* | Electron transport and ATP synthesis | | * | * | * | * | X | 1 |  |  |  |  | √ | √ |
| *petD* | Electron transport and ATP synthesis | | * | * | * | * | X | * |  |  |  |  |  |  |
| *petF* | Electron transport and ATP synthesis | | * | * | * | * | * | X |  |  |  |  |  |  |
| *petG* | Electron transport and ATP synthesis | | X | X | * | * | X | X |  |  |  |  |  |  |
| *petJ* | Electron transport and ATP synthesis | | * | X | X | X | X | X |  |  |  |  |  |  |
| *petL* | Electron transport and ATP synthesis | | X | X | * | * | X | X |  |  |  |  |  |  |
| *petM* | Electron transport and ATP synthesis | | X | * | * | * | * | X |  |  |  |  |  |  |
| *petN* | Electron transport and ATP synthesis | | X | * | * | * | * | X |  |  |  |  |  |  |
| *psaA* | Photosystem I | | * | * | * | * | X | 2 |  |  |  |  |  |  |
| *psaB* | Photosystem I | | * | * | * | * | X | * |  |  |  |  |  |  |
| *psaC* | Photosystem I | | * | * | * | * | 1 | * |  |  | √ |  |  |  |
| *psaD* | Photosystem I | | * | * | * | * | X | 1 |  |  |  |  | √ | √ |
| *psaE* | Photosystem I | | X | * | * | * | * | X |  |  |  |  |  |  |
| *psaF* | Photosystem I | | * | * | * | * | X | * |  |  |  |  |  |  |
| *psaI* | Photosystem I | | * | * | * | * | X | X |  |  |  |  |  |  |
| *psaJ* | Photosystem I | | * | * | * | * | * | * |  |  |  |  |  |  |
| *psaL* | Photosystem I | | X | * | * | * | X | X |  |  |  |  |  |  |
| *psaM* | Photosystem I | | X | * | * | * | X | X |  |  |  |  |  |  |
| *psb28* | Photosystem II | | */* | */* | */* | */* | X | 1/2 |  |  |  |  | √ | √ |
| *psbA* | Photosystem II | | * | * | * | * | X | 1 |  |  |  |  |  |  |
| *psbB* | Photosystem II | | * | * | * | * | X | 1 |  |  |  |  |  |  |
| *psbC* | Photosystem II | | */*/* | */*/* | */*/* | */*/* | X | 1/2/3 |  |  |  |  | √ | √ |
| *psbD* | Photosystem II | | * | * | * | * | * | * |  |  |  |  |  |  |
| *psbE* | Photosystem II | | * | * | * | * | X | * |  |  |  |  |  |  |
| *psbF* | Photosystem II | | * | * | * | * | X | * |  |  |  |  |  |  |
| *psbH* | Photosystem II | | X | * | * | * | X | X |  |  |  |  |  |  |
| *psbI* | Photosystem II | | X | * | * | * | X | X |  |  |  |  |  |  |
| *psbJ* | Photosystem II | | * | * | * | * | X | * |  |  |  |  |  |  |
| *psbK* | Photosystem II | | X | * | * | * | X | X |  |  |  |  |  |  |
| *psbL* | Photosystem II | | * | * | * | * | X | * |  |  |  |  |  |  |
| *psbN* | Photosystem II | | X | * | * | * | * | X |  |  |  |  |  |  |
| *psbT* | Photosystem II | | * | * | * | * | X | * |  |  |  |  |  |  |
| *psbV* | Photosystem II | | * | * | * | * | X | * |  |  |  |  |  |  |
| *psbX* | Photosystem II | | X | * | * | * | X | X |  |  |  |  |  |  |
| *psbY* | Photosystem II | | X | * | * | * | X | * |  |  |  |  |  |  |
| *rbcL* | Carbon assimilation | | * | * | * | * | X | 1 |  |  |  |  | √ | √ |
| *rbcR* | Signal transduction | | X | * | X | * | X | X |  |  |  |  |  |  |
| *rbcS* | Carbon assimilation | | * | * | * | * | X | * |  |  |  |  |  |  |
| *rpl1* | Transcription and translation | | * | * | * | * | X | X |  |  |  |  |  |  |
| *rpl11* | Transcription and translation | | * | * | * | * | X | * |  |  |  |  |  |  |
| *rpl12* | Transcription and translation | | * | X | * | * | X | * |  |  |  |  |  |  |
| *rpl13* | Transcription and translation | | * | * | * | * | X | * |  |  |  |  |  |  |
| *rpl14* | Transcription and translation | | * | * | * | * | * | * |  |  |  |  |  |  |
| *rpl16* | Transcription and translation | | * | * | * | * | * | * |  |  |  |  |  |  |
| *rpl18* | Transcription and translation | | * | * | * | * | X | * |  |  |  |  |  |  |
| *rpl19* | Transcription and translation | | X | * | * | * | X | X |  |  |  |  |  |  |
| *rpl2* | Transcription and translation | | * | * | * | 1 | X | * |  |  |  | √ |  |  |
| *rpl20* | Transcription and translation | | X | * | * | * | * | X |  |  |  |  |  |  |
| *rpl21* | Transcription and translation | | X | * | * | * | X | X |  |  |  |  |  |  |
| *rpl22* | Transcription and translation | | * | * | * | * | X | 1 |  |  |  |  | √ | √ |
| *rpl23* | Transcription and translation | | * | * | * | * | X | X |  |  |  |  |  |  |
| *rpl24* | Transcription and translation | | * | * | * | * | * | * |  |  |  |  |  |  |
| *rpl27* | Transcription and translation | | * | * | * | * | X | * |  |  |  |  |  |  |
| *rpl29* | Transcription and translation | | X | * | * | * | * | X |  |  |  |  |  |  |
| *rpl3* | Transcription and translation | | * | * | * | * | X | * |  |  |  |  |  |  |
| *rpl31* | Transcription and translation | | * | * | * | * | * | * |  |  |  |  |  |  |
| *rpl32* | Transcription and translation | | * | * | * | * | X | X |  |  |  |  |  |  |
| *rpl33* | Transcription and translation | | X | X | X | * | X | X |  |  |  |  |  |  |
| *rpl34* | Transcription and translation | | X | * | * | * | X | * |  |  |  |  |  |  |
| *rpl35* | Transcription and translation | | * | * | * | * | X | X |  |  |  |  |  |  |
| *rpl36* | Transcription and translation | | * | * | * | * | * | * |  |  |  |  |  |  |
| *rpl4* | Transcription and translation | | X | * | * | * | X | X |  |  |  |  |  |  |
|  |  | | *S. natans VIII*  (SN8) | | | *S.natans I*  (SN1) | *S. fluitans III*  (SF3) | |  | Differentiating Polymorphic Sites | | | | |
| Gene | Function | | C3 | C4 | C6 | C2 | C5 | C1 |  | SN8 | SF3 | SN8 vs.  SN1 | SN8 vs.  SF3 | SN1 vs. SF3 |
| *rpl5* | | Transcription and translation | * | * | * | * | X | X |  |  |  |  |  |  |
| *rpl6* | | Transcription and translation | * | * | * | * | X | * |  |  |  |  |  |  |
| *rpl9* | | Transcription and translation | X | * | * | * | X | X |  |  |  |  |  |  |
| *rpoA* | | Transcription and translation | X | X | * | * | X | X |  |  |  |  |  |  |
| *rpoB* | | Transcription and translation | X | * | * | 1 | X | X |  |  |  | √ |  |  |
| *rpoC1* | | Transcription and translation | X | * | * | 1 | X | X |  |  |  | √ |  |  |
| *rpoC2* | | Transcription and translation | X | * | * | 1 | X | X |  |  |  | √ |  |  |
| *rps1* | | Transcription and translation | X | * | * | * | X | X |  |  |  |  |  |  |
| *rps10* | | Transcription and translation | * | X | * | * | X | X |  |  |  |  |  |  |
| *rps11* | | Transcription and translation | * | * | * | * | X | * |  |  |  |  |  |  |
| *rps12* | | Transcription and translation | * | * | * | * | X | 1 |  |  |  |  | √ | √ |
| *rps13* | | Transcription and translation | * | * | * | * | X | * |  |  |  |  |  |  |
| *rps14* | | Transcription and translation | * | * | * | * | X | * |  |  |  |  |  |  |
| *rps16* | | Transcription and translation | * | * | * | * | X | * |  |  |  |  |  |  |
| *rps17* | | Transcription and translation | * | * | * | * | X | 1 |  |  |  |  | √ | √ |
| *rps18* | | Transcription and translation | * | * | * | * | X | * |  |  |  |  |  |  |
| *rps19* | | Transcription and translation | * | * | * | * | * | * |  |  |  |  |  |  |
| *rps2* | | Transcription and translation | X | * | * | * | X | X |  |  |  |  |  |  |
| *rps20* | | Transcription and translation | X | * | * | * | * | X |  |  |  |  |  |  |
| *rps3* | | Transcription and translation | X | * | * | * | X | X |  |  |  |  |  |  |
| *rps4* | | Transcription and translation | X | * | * | * | X | X |  |  |  |  |  |  |
| *rps5* | | Transcription and translation | * | * | * | * | X | X |  |  |  |  |  |  |
| *rps7* | | Transcription and translation | * | * | * | * | * | * |  |  |  |  |  |  |
| *rps8* | | Transcription and translation | X | * | * | * | X | X |  |  |  |  |  |  |
| *rps9* | | Transcription and translation | * | * | * | * | X | 1 |  |  |  |  | √ | √ |
| *secA* | | Protein import | X | * | * | * | X | X |  |  |  |  |  |  |
| *secY* | | Protein import | X | * | * | * | X | X |  |  |  |  |  |  |
| *sufB* | | Fe-S assembly | * | * | * | * | X | X |  |  |  |  |  |  |
| *sufC* | | Fe-S assembly | X | * | * | * | X | X |  |  |  |  |  |  |
| *tatC* | | Protein import | X | * | * | * | X | X |  |  |  |  |  |  |
| *thiG* | | Carbon assimilation | */* | */* | */* | 1/* | X | */2 |  |  |  | √ | √ | √ |
| *thiS* | | Carbon assimilation | X | * | * | * | * | X |  |  |  |  |  |  |
| *trnL(taa) intron* | | - | * | * | * | * | X | * |  |  |  |  |  |  |
| *tsf* | | Transcription and translation | X | * | * | * | X | X |  |  |  |  |  |  |
| *tufA* | | Transcription and translation | * | * | * | * | X | * |  |  |  |  |  |  |
| *ycf12* | | Photosystem II | X | * | * | * | X | X |  |  |  |  |  |  |
| *ycf19* | | Conserved hypothetical genes | * | * | * | 1 | X | X |  |  |  | √ |  |  |
| *ycf3* | | Photosystem I | X | * | * | * | X | X |  |  |  |  |  |  |
| *ycf33* | | Conserved hypothetical genes | X | * | * | * | X | X |  |  |  |  |  |  |
| *ycf34* | | Conserved hypothetical genes | X | * | * | * | X | X |  |  |  |  |  |  |
| *ycf35* | | Conserved hypothetical genes | X | * | * | * | X | X |  |  |  |  |  |  |
| *ycf37* | | Conserved hypothetical genes | X | * | * | * | X | X |  |  |  |  |  |  |
| *ycf39* | | Conserved hypothetical genes | X | * | * | * | X | X |  |  |  |  |  |  |
| *ycf4* | | Photosystem I | X | * | * | * | X | X |  |  |  |  |  |  |
| *ycf41* | | Conserved hypothetical genes | X | * | * | * | X | X |  |  |  |  |  |  |
| *ycf42* | | Conserved hypothetical genes | * | * | * | * | X | X |  |  |  |  |  |  |
| *ycf46* | | Conserved hypothetical genes | X | * | * | * | X | X |  |  |  |  |  |  |
| *ycf47* | | Conserved hypothetical genes | X | * | * | * | X | X |  |  |  |  |  |  |
| *ycf54* | | Conserved hypothetical genes | * | * | * | * | X | X |  |  |  |  |  |  |
| *ycf65* | | Conserved hypothetical genes | * | * | * | * | X | 1 |  |  |  |  | √ | √ |
| *ycf66* | | Conserved hypothetical genes | * | * | * | * | X | * |  |  |  |  |  |  |
| *16S rns-1 rRNA* | | Ribosomal RNA genes | * | * | * | * | X | * |  |  |  |  |  |  |
| *16S rns-2 rRNA* | | Ribosomal RNA genes | * | * | * | * | X | * |  |  |  |  |  |  |
| *23S rnl-1 rRNA* | | Ribosomal RNA genes | 1 | 1 | 1 | 1 | X | X |  | √ |  | √ |  |  |
| *23S rnl-2 rRNA* | | Ribosomal RNA genes | * | * | * | * | X | * |  |  |  |  |  |  |
| *5S rrn5-1 rRNA* | | Ribosomal RNA genes | * | * | * | * | * | * |  |  |  |  |  |  |
| *5S rrn5-2 rRNA* | | Ribosomal RNA genes | * | * | * | * | X | * |  |  |  |  |  |  |
| *trnA-1(ugc) tRNA* | | Transfer RNA genes | * | * | * | * | * | * |  |  |  |  |  |  |
| *trnA-2(ugc) tRNA* | | Transfer RNA genes | * | * | * | * | * | * |  |  |  |  |  |  |
| *trnC(gca) tRNA* | | Transfer RNA genes | * | * | * | * | X | * |  |  |  |  |  |  |
| *trnD(guc) tRNA* | | Transfer RNA genes | * | * | * | * | X | * |  |  |  |  |  |  |
| *trnE(uuc) tRNA* | | Transfer RNA genes | * | * | * | * | X | * |  |  |  |  |  |  |
| *trnF(gaa) tRNA* | | Transfer RNA genes | * | X | * | * | X | * |  |  |  |  |  |  |
| *trnG-1(gcc) tRNA* | | Transfer RNA genes | * | * | * | * | X | 1 |  |  |  |  | √ | √ |
| *trnG-2(ucc) tRNA* | | Transfer RNA genes | * | * | * | * | * | * |  |  |  |  |  |  |
| *trnH(gug) tRNA* | | Transfer RNA genes | * | * | * | * | * | * |  |  |  |  |  |  |
| *trnI-1(gau) tRNA* | | Transfer RNA genes | * | * | * | * | X | * |  |  |  |  |  |  |
| *trnI-2(gau) tRNA* | | Transfer RNA genes | * | * | * | * | 1 | * |  |  | √ |  | √ | √ |
| *trnK(uuu) tRNA* | | Transfer RNA genes | * | * | * | * | * | * |  |  |  |  |  |  |
| *trnL-1(uaa) tRNA* | | Transfer RNA genes | * | * | * | * | X | * |  |  |  |  |  |  |
| *trnL-2(uag) tRNA* | | Transfer RNA genes | X | * | * | * | X | X |  |  |  |  |  |  |
| *trnM-1(cau) tRNA* | | Transfer RNA genes | * | X | * | 1 | X | * |  |  |  | √ |  | √ |
| *trnM-2(cau) tRNA* | | Transfer RNA genes | * | * | * | * | X | * |  |  |  |  |  |  |
| *trnM-3(cau) tRNA* | | Transfer RNA genes | * | * | * | * | X | * |  |  |  |  |  |  |
| *trnN(guu) tRNA* | | Transfer RNA genes | * | * | * | * | X | * |  |  |  |  |  |  |
| *trnP(ugg) tRNA* | | Transfer RNA genes | * | * | * | * | X | * |  |  |  |  |  |  |
| *trnQ(uug) tRNA* | | Transfer RNA genes | * | * | * | * | * | * |  |  |  |  |  |  |
| *trnR-1(acg) tRNA* | | Transfer RNA genes | * | * | * | * | * | * |  |  |  |  |  |  |
| *trnR-2(ucu) tRNA* | | Transfer RNA genes | * | * | * | * | * | * |  |  |  |  |  |  |
| *trnS-1(gcu) tRNA* | | Transfer RNA genes | * | X | * | * | X | * |  |  |  |  |  |  |
| *trnS-2(uga) tRNA* | | Transfer RNA genes | * | * | * | * | X | * |  |  |  |  |  |  |
| *trnT(ugu) tRNA* | | Transfer RNA genes | * | * | * | 1 | X | * |  |  |  | √ |  | √ |
| *trnV(uac) tRNA* | | Transfer RNA genes | * | * | * | * | X | * |  |  |  |  |  |  |
| *trnW(cca) tRNA* | | Transfer RNA genes | X | X | X | X | X | X |  |  |  |  |  |  |
| *trnY(gua) tRNA* | | Transfer RNA genes | * | * | * | * | * | * |  |  |  |  |  |  |
